# Activation of β- and γ-carbonic anhydrases from pathogenic bacteria with tripeptides

**DOI:** 10.1080/14756366.2018.1468530

**Published:** 2018-05-11

**Authors:** Azzurra Stefanucci, Andrea Angeli, Marilisa Pia Dimmito, Grazia Luisi, Sonia Del Prete, Clemente Capasso, William A. Donald, Adriano Mollica, Claudiu T. Supuran

**Affiliations:** a Department of Pharmacy, “Gabriele d’Annunzio” University of Chieti-Pescara, Chieti, Italy;; b Department of Neurofarba, Università degli Studi di Firenze, Sezione di Scienze Farmaceutiche e Nutraceutiche, Sesto Fiorentino, Florence, Italy;; c Istituto di Bioscienze e Biorisorse, CNR, Napoli, Italy;; d School of Chemistry, University of New South Wales, Sydney, New South Wales, Australia

**Keywords:** Carbonic anhydrase, tripeptide, activator, proton transfer, pathogenic bacteria

## Abstract

Six tripeptides incorporating acidic amino acid residues were prepared for investigation as activators of β- and γ-carbonic anhydrases (CAs, EC 4.2.1.1) from the pathogenic bacteria *Vibrio cholerae, Mycobacterium tuberculosis,* and *Burkholderia pseudomallei*. The primary amino acid residues that are involved in the catalytic mechanisms of these CA classes are poorly understood, although glutamic acid residues near the active site appear to be involved. The tripeptides that contain Glu or Asp residues can effectively activate VchCAβ and VchCAγ (enzymes from *V. cholerae*), Rv3273 CA (mtCA3, a β-CA from *M. tuberculosis*) and BpsCAγ (γ-CA from *B. pseudomallei*) at 0.21–18.1 µM levels. The position of the acidic residues in the peptide sequences can significantly affect bioactivity. For three of the enzymes, tripeptides were identified that are more effective activators than both l-Glu and l-Asp. The tripeptides are also relatively selective because they do not activate prototypical α-CAs (human carbonic anhydrases I and II). Because the role of CA activators in the pathogenicity and life cycles of these infectious bacteria are poorly understood, this study provides new molecular probes to explore such processes.

## Introduction

Carbonic anhydrases (CAs, EC 4.2.1.1) are ubiquitous enzymes that interconvert carbon dioxide and bicarbonate^1–9^. There are seven genetically distinct CA families known to date in organisms across the phylogenetic tree. The α-CAs are widespread in vertebrates (in the form of a multitude of isoforms, including 15 in humans)^1^^,^^2^, prokaryotes, and simpler eukaryotes (such as protozoa, fungi and some bacteria)^3–6^. The β- and γ-class enzymes are widespread in bacteria and archaea, but are not found in eukaryotic organisms^7–9^. CA inhibitors (CAIs) show pharmacologic applications in pathologies in which the activity of these enzymes is dysregulated (in humans), such as edema^10^, glaucoma^11^, neurologic diseases (epilepsy, etc.)^12^, obesity^13^, and some tumors^14^, and many sulphonamide or sulphamate CAIs have been in clinical use for decades^15^. In contrast, investigation of activators of these enzymes (CAAs) have been relatively limited^2^. Recently, the potential to use of CAAs as pharmacological agents for pathologies related to cognitive impairment has been demonstrated, which may result in innovative memory therapies^16^.

The CA catalytic mechanism is represented by Equations (1) and (2), where “E” corresponds to enzyme.

$${\text{H}}_{2}\text{O}$$

(1) $${\text{EZn}}^{\text{2}+}-{\text{OH}}^{-}+{\text{CO}}_{\text{2}}\Leftrightarrow {\text{EZn}}^{\text{2}+}-{\text{HCO}}_{\text{3}}^{-}\Leftrightarrow {\text{EZn}}^{\text{2}+}-{\text{OH}}_{\text{2}}+{\text{HCO}}_{\text{3}}^{-}$$

(2) $${\text{EZn}}^{\text{2}+}-{\text{OH}}_{\text{2}}\Leftrightarrow {\text{EZn}}^{\text{2}+}-{\text{HO}}^{-}+{\text{H}}^{+}$$

First, a reactive metal hydroxide species nucleophilically attacks a CO_2_ molecule that is bound in a hydrophobic pocket within the active site of the enzyme to form a metal-bound bicarbonate^17^. Typically, zinc is the metal ion in the active sites of most CA classes, although Cd(II) and Fe(II) may also work for some CAs^1^. The bicarbonate can be readily replaced by an incoming water molecule to generate an acidic metal-bound water molecule. In the rate-determining step, a proton is transferred from the metal-coordinated water molecule to the reaction medium to reform the metal-hydroxide species^18^^,^^19^. In all CA classes that have been investigated in detail to date, the rate-determining step is assisted by amino acid residues that are positioned in the active site pocket to favor the proton-transfer process^1–4^^,^^18–21^. For α-CAs that have been the most extensively studied CAs, the proton shuttling residue is a His placed in the middle of the active site pocket (His64, CA I numbering system)^18^. However, this proton-transfer process is less well understood in all other CA classes. For β-CAs, His and Tyr residues (His92 and Tyr88, *Coccomyxa* CA numbering)^20^ may act as proton shuttle residues. For γ-CAs, Ferry’s group^21^ reported that one or two Glu residues (Glu84 and Glu62, Cam numbering system; Cam is the enzyme from *Methanosarcina thermophila*)^21^ act as proton shuttles in the catalytic cycle. In the presence of activators (A in Equation (3), enzyme-activator complexes can be formed^19^, and the proton transfer reaction is intramolecular and more efficient than intermolecular transfer to buffer molecules, which conventionally occurs in the absence of activators (Equation (3)).

(3) $${\text{EZn}}^{\text{2}+}-{\text{OH}}_{\text{2}}+\text{A}\Leftrightarrow [{\text{EZn}}^{\text{2}+}-{\text{OH}}_{\text{2}}-\text{A}]\Leftrightarrow [{\text{EZn}}^{\text{2}+}-{\text{HO}}^{-}-{\text{AH}}^{+}]\Leftrightarrow {\text{EZn}}^{\text{2}+}-{\text{HO}}^{-}+{\text{AH}}^{+}\;\text{enzyme–activator complexes}$$

CAAs have been investigated in detail for human (h) α-CAs, by means of X-ray crystallography, kinetic and spectroscopic methods^19^^,^^22–24^, and several drug design studies have also been reported^24–27^. However, no drug-design CAA studies are available for bacterial, β- and γ-CAs. These enzymes have only recently been started to be investigated for their activation with amines and amino acids^28–31^. The design of bioactive molecules that modulate these enzymes may be useful for controlling the intra- and extracellular pH of microorganisms, which can play crucial roles in the life cycles of pathogenic microorganisms. Here, we report such a study for investigating whether tripeptides incorporating acidic amino acid residues do show activating effects against β- and γ-class CAs from pathogenic bacteria such as *Vibrio cholerae* (the enzymes included in the study were VchCAβ and VchCAγ), *Mycobacterium tuberculosis* (Rv3273, also called mtCA3, one of the three β-CAs from this bacterium was considered here) and *Burkholderia pseudomallei* (BpsCAγ, a γ-CA from this pathogenic organism was used for our investigations)^28–31^. The amino acids used for obtaining these tripeptides, apart for the acidic ones (Glu and Asp) included aromatic (His, Phe and Tyr), hydroxy (Ser and Thr) as well as aliphatic (Ile) residues, in order to investigate the role that such structural elements may induce to the CA activating effects.

## Materials and methods

### Chemistry

All solvents and coupling reagents were purchased from VWR (Radnor, PN, USA). Fmoc amino acids and Fmoc-Rink-amide MBHA resin (0.68 mmol/g) were purchased from Chem-Impex (Wood Dale, IL, USA) and IRIS Biotech GmbH (Marktredwitz, DH, Germany) respectively. O*t*Bu was chosen as orthogonal protection on Tyr, Thr, Ser, Asp and Glu side chains, Boc protecting group for His side chain and Trt for Asn and Gln side chains. The peptides were synthesized by Fmoc-SPPS (standard solid phase peptide synthesis) using TBTU/HOBt for coupling reactions and piperidine 20% solution in DMF for Fmoc group deprotection as previously described^32^.

Purification of compounds was carried out by RP-HPLC using a Waters XBridge Prep BEH130 C18, 5.0 μm, 250 × 10 mm column at a flow rate of 4.0 ml/min on a Waters Binary pump 1525, and a linear gradient of H_2_O/acetonitrile 0.1% TFA ranging from 5% acetonitrile to 95% acetonitrile for 35 min. The purity of all final TFA salts was confirmed by NMR analysis, ESI-LRMS, and analytical RP-HPLC (C18-bonded 4.6 × 150 mm) at a flow rate of 1 ml/min, using as eluent a gradient of H_2_O/acetonitrile 0.1% TFA ranging from 5% acetonitrile to 95% acetonitrile in 30 min and was found to be ≥95% (*R*_t_ reported in Table 1). Nuclear magnetic resonance (NMR) spectra of the final compounds were recorded on a Varian Inova 300 MHz spectrometer using DMSO-d6 as solvent. The mass spectrometry (MS) system used consisted of an LCQ (Thermo Finnigan) ion trap mass spectrometer (San Jose, CA, USA) equipped with an electrospray ionization (ESI) source. The capillary temperature was set at 300 °C and the spray voltage at 4.00 kV. The fluid was nebulized using nitrogen (N_2_) as the sheath and auxiliary.

**Table 1.** Characterization data for the new peptides^.^NH_2_-Xaa_1_-Xaa_2_-Xaa_3_-NH_2_**1**–**6** (TFA salts).

Compounds	Xaa_1_	Xaa_2_	Xaa_3_	*R*_t_ (HPLC)^a^ min.	MS calcd.	MS found
**1**	Tyr	Phe	Asp	12.44	442.19	443.31
**2**	His	Phe	Glu	11.57	430.21	431.41
**3**	Glu	Ile	Thr	11.78	360.41	361.56
**4**	Gln	Asp	Ser	11.11	347.14	348.25
**5**	Asn	Asp	Ser	11.08	333.13	334.18
**6**	Glu	Phe	Glu	11.70	422.43	423.51

^a^Analytical RP HPLC: C18 linear gradient of H_2_O/acetonitrile 0.1% TFA starting from 5% acetonitrile to 95% acetonitrile in 30 min (solvent ramp: from 0 to 5 min: 5% ACN; from 5 to 20 min: 80% ACN; from 20 min to 25 min: 20% ACN; from 25 min to 30 min: 5% ACN).

### General procedures for the tripeptide synthesis

#### Loading of the first amino acid

The resin was treated with a 20% piperidine solution in DMF (2*15 min) and then washed with DMF/MeOH/DCM. Then, the Fmoc protected amino acid (3 equiv) was dissolved in DMF (3 ml). TBTU (3 equiv) and DIPEA (6 equiv) were added and the resulting mixture was added to the resin. The Kaiser test was used to check the reaction. When complete, the resin was washed with DMF/MeOH/DCM.

#### Amino acids couplings

3 Equiv. of amino acid was dissolved in DMF (3 ml) together with TBTU (3 equiv.) and DIPEA (6 equiv.). Then the resulting mixture was added to the resin. The Kaiser test was used to check the reaction. When complete, the resin was washed with DMF/MeOH/DCM.

#### Cleavage and purification

The resin was treated with TFA/H_2_O/TIPS 95:2.5:2.5 (5 ml for 1 h) and filtered. The solution was concentrated to 1 ml and precipitated in 10 ml of cold Et_2_O. The suspension was centrifuged and washed three times with fresh Et_2_O. The crude solid was dried in high vacuum and purified on RP-HPLC.

##### Characterization data for new compounds

TFA·NH_2_-Tyr-Phe-Asp-NH_2_ (**1**): 81% yield; *R*_t_ (HPLC) = 12.44 min. ^1^H NMR (DMSO-d_6_) δ: 8.78 (1H, d, NH Phe), 8.44 (1H, d, NH Asp), 7.92 (3H, s, NH_3_^+^), 7.26–7.16 (7H, m, NH_2_ Asp + 5H Phe aromatics), 7.14 (2H, s, NH_2_ Asp amide), 7.03 (2H, dd, Tyr aromatics), 6.66 (2H, dd, Tyr aromatics), 4.58–4.52 (2H, m, CH^α^ Phe + CH^α^ Tyr), 3.85 (1H, m, CH^α^ Asp), 3.04 (1H, dd, CH^β^ Phe), 2.98–2.52 (1H, dd, CH^β^ Phe + 2H, m, CH_2_^β^ Tyr); MS calcd: 442.19, found: 443.31.

TFA^.^NH_2_-His-Phe-Glu-NH_2_ (**2**): 57% yield; *R*_t_ (HPLC) = 11.57 min. ^1^H NMR (DMSO-d_6_) δ: 8.80 (3H, s, NH_3_^+^), 8.64 (1H, d, NH Phe), 7.32 (1H, d, NH Glu), 7.24 (1H, s, CH^τ^ His), 7.15 (2H, s, NH_2_ Glu amide), 7.07 (1H, d, NH His), 6.90 (1H, d, CH^π^ His), 4.59 (1H, m, CH^α^ Phe), 4.20 (1H, m, CH^α^ His), 3.96 (1H, m, CH^α^ Glu), 3.08–2.83 (2H, m, CH_2_^β^ Phe), 2.73–2.52 (2H, m, CH_2_^β^ His), 2.41–2.20 (2H, m, CH_2_^β^ Glu), 1.81–1.68 (2H, m, CH_2_^γ^ Glu). MS calcd: 430.21, found: 431.41.

TFA^.^NH_2_-Glu-Ile-Thr-NH_2_ (**3**): 83% yield; *R*_t_ (HPLC) = 11.78 min. ^1^H NMR (DMSO-d_6_) δ: 8.54 (1H, d, NH Thr), 8.12 (3H, s, NH_3_^+^), 7.92 (2H, s, NH Ile), 7.11 (2H, s, NH_2_ Thr amide), 4.15 (1H, d, CH^α^ Ile), 4.11 (1H, d, CH^α^ Thr), 3.98 (1H, quint, CH^β^ Thr), 3.85 (1H, m, CH^α^ Glu), 2.27 (2H, t, CH_2_^β^ Glu), 1.87 (2H, m, CH_2_^γ^ Glu), 1.72 (1H, m, CH^β^ Ile), 1.43 (1H, m, CH^γ^ Ile), 1.07 (1H, m, CH^γ^ Ile), 0.96 (3H, d, CH_3_ Thr), 0.94 (3H, d, CH_3_ Ile), 0.84 (3H, t, CH_3_^δ^ Ile). MS calcd: 360.41, found: 461.56.

TFA^.^NH_2_-Gln-Asp-Ser-NH_2_ (**4**): 87% yield; *R*_t_ (HPLC) = 11.11 min. ^1^H NMR (DMSO-d_6_) δ: 8.79 (1H, d, NH Asp), 8.15 (3H, s, NH_3_^+^), 7.98 (1H, d, NH Ser), 7.17 (2H, s, NH_2_ Gln), 7.11 (2H, s, NH_2_ Ser amide), 4.64 (1H, m, CH^α^ Asp), 4.13 (1H, m, CH^α^ Ser), 3.52 (1H, m, CH^α^ Gln), 2.75–2.56 (2H, m, CH_2_^β^ Asp and CH_2_^β^ Ser), 2.21 (2H, t, CH_2_^β^ Gln), 1.91 (2H, m, CH_2_^γ^ Gln). MS calcd: 347.14, found: 348.25.

TFA^.^NH_2_-Asn-Asp-Ser-NH_2_ (**5**): 84% yield; *R*_t_ (HPLC) = 11.08 min. ^1^H NMR (DMSO-d_6_) δ: 8.76 (1H, d, NH Asp), 8.08 (3H, s, NH_3_^+^), 7.94 (1H, d, NH Ser), 7.71 (1H, s, NH_2_ Asn), 7.31 (1H, s, NH_2_ Asn), 7.15 (2H, s, NH_2_ Ser amide), 4.61 (1H, m, CH^α^ Asp), 4.14–3.62 (4H, m, CH^α^ Ser, CH^α^ Asn, CH^α^ Ser and OH Ser broad singlet at 3.863), 2.73 (2H, m, CH_2_^β^ Asp), 2.69–2.48 (4H, m, CH_2_^β^ Asn, CH_2_^β^ Ser). MS calcd: 333.13, found: 334.18.

TFA^.^NH_2_-Glu-Phe-Glu-NH_2_ (**6**): 77% yield; *R*_t_ (HPLC) = 11.70 min. ^1^H NMR (DMSO-d_6_) δ: 8.61 (1H, d, NH Phe), 8.32 (1H, d, NH Glu), 8.05 (3H, s, NH_3_^+^ Glu), 7.31–7.19 (5H, m, Phe aromatics), 7.08 (2H, s, NH_2_ Glu amide), 4.58 (1H, m, CH^α^ Phe), 4.19 (1H, m, CH^α^ Glu), 3.73 (1H, m, CH^α^ Glu), 3.07 (1H, dd, CH^β^ Phe), 2.75 (1H, dd, CH^β^ Phe), 2.31–2.23 (4H, m, 2CH_2_^β^ Glu), 1.93–1.76 (4H, m, 2CH_2_^γ^ Glu). MS calcd: 422.43, found: 423.51.

### CA enzyme activation assay

An Sx.18Mv-R Applied Photophysics (Oxford, UK) stopped-flow instrument has been used to assay the catalytic activity of various CA isozymes for CO_2_ hydration reaction^33^. Phenol red (at a concentration of 0.2 mM) was used as indicator, working at the absorbance maximum of 557 nm, with 10 mM Hepes (pH 7.5) or TRIS (pH 8.3) as buffers, 0.1 M Na_2_SO_4_ (for maintaining constant ionic strength), following the CA-catalysed CO_2_ hydration reaction for a period of 10 s at 25 °C. Activity of the α-CAs was measured at pH 7.5 whereas that of the β-class enzymes at pH 8.3 in order to avoid the possibility that their active site is closed^34^. The CO_2_ concentrations ranged from 1.7 to 17 mM for the determination of the kinetic parameters and activation constants. For each activator at least six traces of the initial 5–10% of the reaction have been used for determining the initial velocity. The uncatalysed rates were determined in the same manner and subtracted from the total observed rates. Stock solutions of activators (10 mM) were prepared in distilled-deionized water and dilutions up to 1 nM were done thereafter with the assay buffer. Activator and enzyme solutions were pre-incubated together for 15 min (standard assay at room temperature) prior to assay, in order to allow for the formation of the E–A complex. The activation constant (K_A_), defined similarly with the inhibition constant K_I_, can be obtained by considering the classical Michaelis–Menten equation (Equation (4), which has been fitted by non-linear least squares by using PRISM 3:

(4) $$v=v_{\text{max}}/\left\{\text{1}+{\text{K}}_{\text{M}}/\left[\text{S}\right]\left(\text{1}+{\left[\text{A}\right]}_{\text{f}}/{\text{K}}_{\text{A}}\right)\right\}$$

where [A]_f_ is the free concentration of activator.

Working at substrate concentrations considerably lower than K_M_ ([S] **≪ **K_M_), and considering that [A]_f_ can be represented in the form of the total concentration of the enzyme ([E]_t_) and activator ([A]_t_), the obtained competitive steady-state equation for determining the activation constant is given by Equation (5):

(5) $$v=v_{0}\cdot {\text{K}}_{\text{A}}/\left\{{\text{K}}_{\text{A}}+{\left({\left[\text{A}\right]}_{\text{t}}-0.\text{5}\{\left({\left[\text{A}\right]}_{\text{t}}+{\left[\text{E}\right]}_{\text{t}}+{\text{K}}_{\text{A}}\right)-{\left({\left[\text{A}\right]}_{\text{t}}+{\left[\text{E}\right]}_{\text{t}}+{\text{K}}_{\text{A}}\right)}^{\text{2}}-\text{4}{\left[\text{A}\right]}_{\text{t}}\cdot {\left[\text{E}\right]}_{\text{t}}\right)}^{\text{1}/\text{2}}\right\}\}$$

where *v*_0_ represents the initial velocity of the enzyme-catalysed reaction in the absence of activator^35–38^.

## Results and discussion

### Chemistry

Here, we designed the tripeptides **1**–**6** with amidated C-termini that incorporate at least one acidic amino acid (Asp and Glu) residue at various positions in the sequence (Table 1). In the sequence assemblage, we focused on our previous observation that single acidic amino acids like Asp and Glu act as powerful activators of selected bacterial CAs, in view of the fact that both possess the –COO^−^ functionality that can participate in the proton transfer process^28–31^. Aromatic amino acids such as Phe and Tyr were also found to have significant activating abilities on the CAs belonging to pathogenic bacteria^28–31^. Thus, we considered three groups of compounds: (i) a pair of tripeptides that share the terminal dipeptide motif Asp-Ser, i.e. H-Gln-Asp-Ser-NH_2_ (**4**) and H-Asn-Asp-Ser-NH_2_ (**5**); (ii) H-Tyr-Phe-Asp-NH_2_ (**1**), H-His-Phe-Glu-NH_2_ (**2**), and H-Glu-Phe-Glu-NH_2_ (**6**), all containing a central Phe, and characterized by Tyr, His, and Glu, respectively, as amino-terminals, and Glu or Asp at the carboxy-end; and (iii) H-Glu-Ile-Thr-NH_2_ (**3**), which features a Glu residue at the N-terminal position. Peptides **1**–**6** were efficiently synthesized by following routine SPPS procedures^32^, and obtained in the amidated form as TFA salts. Their main analytical data (HPLC and MS) are reported in Table 1. The complete characterization is shown in section 2.

### CA activation studies

The six peptides activated the enzymes from pathogenic bacteria investigated here (Table 2), i.e. the β- and γ-CAs from *V. cholerae* (VchCAβ and VchCAγ), the Rv3273 CA (also called mtCA3, a β-CA from *M. tuberculosis*) and BpsCAγ (a γ-CA from *B. pseudomallei*). These four pathogens produce serious diseases in humans, and understanding factors connected to their invasion, colonization and virulence, and how these factors are influenced by modulators of CA activity, may be relevant to developing new therapeutic strategies devoid of the extensive drug resistance that has ultimately emerged for most clinically used anti-infective drugs^28–31^.

**Table 2.** Activation of hCA I, hCA II, VchCAβ, Rv3273, VchCAγ and BpsCAγ with tripeptides **1**–**6** and simple amino acid derivatives, by a stopped-flow, CO_2_ hydrase assay, at 25 °C and pH 8.4^33^.

K_A_ (µM)*						
Activator	hCA I^a^	hCA II^a^	VchCAβ	Rv3273	VchCAγ	BpsCAγ
**1**	>50	>50	3.52 ± 0.18	8.45 ± 0.11	14.7 ± 0.21	10.1 ± 0.09
**2**	>50	>50	1.16 ± 0.05	6.29 ± 0.14	5.84 ± 0.15	1.63 ± 0.12
**3**	>50	>50	1.15 ± 0.10	4.32 ± 0.08	11.9 ± 0.42	3.75 ± 0.15
**4**	>50	>50	0.21 ± 0.07	15.8 ± 0.76	12.9 ± 0.61	6.18 ± 0.30
**5**	>50	>50	7.16 ± 0.34	18.1 ± 1.02	10.6 ± 0.74	0.95 ± 0.09
**6**	>50	>50	4.18 ± 0.23	9.40 ± 0.62	2.74 ± 0.16	5.24 ± 0.30
l-Asp	5.20	>50	9.87 ± 0.41	10.1 ± 0.84	8.95 ± 0.46	10.7 ± 0.85
l-Asn	11.3	>50	>50	10.0 ± 0.71	6.37 ± 0.29	0.98 ± 0.08
l-Glu	6.43	>50	0.69 ± 0.05	>50	6.48 ± 0.50	3.25 ± 0.17
l-Gln	>50	>50	18.1 ± 0.94	21.6 ± 1.1	9.21 ± 0.82	6.15 ± 0.30
l-His^a^	0.03	10.9	20.3	18.2	1.01	24.7
l-Phe^a^	0.07	0.013	15.4	30.6	0.73	1.73

*Mean ± standard error, from three different assays.

^a^From Refs.^28–31^.

In Table 2, the activation constants of tripeptides **1**–**6**, and some amino acids for four bacterial enzymes and the ubiquitous isoforms hCA I and II are shown. The six amino acids were included in this study for comparative reasons. The activation hCA I and II by these six amino acids were measured previously^23^^,^^24^. The activation constants of l-Phe and l-His for the four enzymes from pathogenic bacteria were also recently reported^28–31^.

The following structure–activity relationship (SAR) can be obtained from the data in Table 2:

(i) VchCAβ was effectively activated by tripeptides **1**–**6** with activation constants ranging between 0.21 and 7.16 µM. The most effective activator was **4** (GlnAspSer), whereas the least effective one was **5** (AsnAspSer). Thus, the extra methylene group in Gln compared to Asn resulted in tripeptide **4** more effectively activating this enzyme by 34 times compared to **5** (Table 2). Other effective activators against this enzyme include tripeptides **2** and **3** that incorporate a Glu residue in the sequence. However, the tripeptide with two Glu residues (**6**) was less effective as an activator compared to **2** and **3**. It is interesting that l-Glu is a very effective VchCAβ activator (K_A_ of 0.69 µM), whereas l-Gln, l-His and l-Phe are much less effective activators (Table 2). l-Asp is moderately potent as an activator (K_A_ of 9.87 µM), but l-Asn is not.

(ii) The other β-CA investigated here, Rv3273, was less sensitive to these activators compared to that from *V. cholerae* enzyme; i.e. tripeptides **1**–**6** had K_A_s in the range of 4.32 to 18.1 µM for this CA. The most effective activator was **3** (GluIleThr), whereas the least effective was **5** (AsnAspSer). Tripeptide **2** was the next most effective activator after **3**. These latter two peptides both have one Glu residue, albeit in opposing positions (amino-terminal vs carboxy-terminal). Considering the simple amino acid derivatives of Table 2, l-Glu was in this case ineffective as an activator whereas the remaining amino acids were moderately potent to weak activators (activation constants from 10.0 to 30.6 µM).

(iii) VchCAγ was activated by tripeptides **1**–**6** with K_A_s ranging between 2.74 and 14.7 µM. The most effective activator was **6**, which incorporates two Glu residues in the sequence, followed by **2**, which has one such carboxy-terminal residue. The remaining tripeptides were less effective activators, with K_A_s > 10 µM (Table 2). For this isoform, the best activators were the simple aromatic amino acids l-His and l-Phe (K_A_s of 0.73–1.01 µM) whereas l-Asp, l-Asn, l-Glu and l-Gln showed activities in the range of 6.37–9.21 µM. Thus, the SAR is rather challenging to delineate for this enzyme and with this series of activators.

(iv) BpsCAγ was efficiently activated by tripeptides **1**–**6** with K_A_s ranging between 0.95 and 10.1 µM. The best activators were **5** and **2** (K_A_s of 0.95 and 1.63 µM, respectively), which do not share much in similarity except that in both sequences there is one acidic amino acid residue, Asp in **5**, and Glu in **2**. The most ineffective activator was **1**, which does not incorporate such a residue. However, it is interesting to note that l-Asn with a K_A_ of 0.98 µM was the most effective activator among the simple amino acids considered in the study. Indeed, this latter activation constant was one order of magnitude lower than that for l-Asp, whereas such an important difference is not seen for the l-Glu/l-Gln pair (Table 2).

(v) A very interesting observation is the fact that the human isoforms hCA I and II were not at all activated by tripeptides **1**–**6** investigated here (K_A_ > 50 µM), although they are highly activated by some of the amino acids, such as l-His, and l-Phe. hCA II is in fact sensitive only to these two amino acids, whereas hCA I is also activated by l-Asp, l-Asn, l-Glu (but not l-Gln) and of course, l-His and l-Phe (there are X-ray crystal structures for adducts of hCA I/II with some of these two amino acids, which proved in detail the activation mechanism of α-Cas)^23^^,^^24^.

## Conclusions

We discovered a very interesting class of tripeptide activators for bacterial β- and γ-class CAs, which do not interfere with the activity of the off-target, human isoforms hCA I and II. These activators incorporate aromatic amino acid residues, as well as acidic (Asp and Glu) residues in their sequence which may have roles in the rate-determining proton-transfer processes in the catalytic mechanism of these enzymes. The activity of the tripeptides differ both across the two classes of enzymes and between particular members of each class from different pathogens, such as *V. cholerae, M. tuberculosis* and *B. pseudomallei*. Overall, these tripeptides may be useful as tools for investigating the role of these enzymes in key bacterial processes such as invasion, colonization and pathogenicity, which are currently poorly understood.

## References

[1] a) Supuran CT. Structure and function of carbonic anhydrases. Biochem J 2016;473:2023–32. b) Supuran CT. Carbonic anhydrases and metabolism. Metabolites 2018;8:E25. 10.1042/BCJ20160115 27407171

[2] Supuran CT. Carbonic anhydrases: novel therapeutic applications for inhibitors and activators. Nat Rev Drug Discov 2008;7:168–81. 18167490 10.1038/nrd2467

[3] a) Supuran CT. How many carbonic anhydrase inhibition mechanisms exist? J Enzyme Inhib Med Chem 2016;31:345–60. b) Supuran CT, Alterio V, Di Fiore A, et al Inhibition of carbonic anhydrase IX targets primary tumors, metastases and cancer stem cells: three for the price of one. Med Res Rev 2018, (doi: 10.1002/med.21497); 26619898

[4] a) Capasso C, Supuran CT. An overview of the alpha-, beta-and gamma-carbonic anhydrases from Bacteria: can bacterial carbonic anhydrases shed new light on evolution of bacteria? J Enzyme Inhib Med Chem 2015;30:325–32. b) Supuran CT, Capasso C, New light on bacterial carbonic anhydrases phylogeny based on the analysis of signal peptide sequences. J Enzyme Inhib Med Chem 2016;3:1254–60. c) Supuran CT, Capasso C. Carbonic anhydrase from *Porphyromonas gingivalis* as a drug target. Pathogens 2017;6:E30 d) Maresca A, Carta F, Vullo D, Supuran CT. Dithiocarbamates strongly inhibit the β-class carbonic anhydrases from *Mycobacterium tuberculosis*. J Enzyme Inhib Med Chem 2013;28:407–11. e) Chohan ZH, Arif M, Shafiq Z, et al In vitro antibacterial, antifungal & cytotoxic activity of some isonicotinoylhydrazide Schiff’s bases and their cobalt (II), copper (II), nickel (II) and zinc (II) complexes. J Enzyme Inhib Med Chem 2006;21:95–103.

[5] a) Del Prete S, Vullo D, Fisher GM, et al Discovery of a new family of carbonic anhydrases in the malaria pathogen *Plasmodium falciparum* – the η-carbonic anhydrases. Bioorg Med Chem Lett 2014;24:4389–96. b) Del Prete S, De Luca V, De Simone G, et al Cloning, expression and purification of the complete domain of the η-carbonic anhydrase from *Plasmodium falciparum*. J Enzyme Inhib Med Chem 2016;31:54–9. c) De Menezes Dda R, Calvet CM, Rodrigues GC, et al Hydroxamic acid derivatives: a promising scaffold for rational compound optimization in Chagas disease. J Enzyme Inhib Med Chem 2016;31:964–73.

[6] Xu Y, Feng L, Jeffrey PD, et al Structure and metal exchange in the cadmium carbonic anhydrase of marine diatoms. Nature 2008;452:56–61. 18322527 10.1038/nature06636

[7] a) Zimmerman SA, Ferry JG, Supuran CT. Inhibition of the archaeal β-class (Cab) and γ-class (Cam) carbonic anhydrases. Curr Top Med Chem 2007;7:901–8. b) De Simone G, Supuran CT. (In)organic anions as carbonic anhydrase inhibitors. J Inorg Biochem 2012;111:117–29. 10.2174/156802607780636753 17504135

[8] a) Schlicker C, Hall RA, Vullo D, et al Structure and inhibition of the CO_2_-sensing carbonic anhydrase Can2 from the pathogenic fungus *Cryptococcus neoformans*. J Mol Biol 2009;3:1207–20. b) Ozensoy Guler O, Capasso C, Supuran CT. A magnificent enzyme superfamily: carbonic anhydrases, their purification and characterization. J Enzyme Inhib Med Chem 2016;31:689–94. c) De Simone G, Langella E, Esposito D, et al Insights into the binding mode of sulphamates and sulphamides to hCA II: crystallographic studies and binding free energy calculations. J Enzyme Inhib Med Chem 2017;32:1002–11.

[9] a) Nocentini A, Cadoni R, Del Prete S, et al Benzoxaboroles as efficient inhibitors of the β-carbonic anhydrases from pathogenic fungi: activity and modeling study. ACS Med Chem Lett 2017;8:1194–8. b) Supuran CT, Scozzafava A, Mastrolorenzo A. Bacterial proteases: current therapeutic use and future prospects for the development of new antibiotics. Expert Opin Pat 2001;11:221–59. c) Del Prete S, De Luca V, Vullo D, et al A new procedure for the cloning, expression and purification of the β-carbonic anhydrase from the pathogenic yeast *Malassezia globosa*, an anti-dandruff drug target. J Enzyme Inhib Med Chem 2016;31:1156–61. 10.1021/acsmedchemlett.7b00369 PMC5682618 29152053

[10] a) Carta F, Supuran CT. Diuretics with carbonic anhydrase inhibitory action: a patent and literature review (2005–2013). Expert Opin Ther Pat 2013;23:681–91. b) Menchise V, De Simone G, Alterio V, et al Carbonic anhydrase inhibitors: stacking with Phe131 determines active site binding region of inhibitors as exemplified by the X-ray crystal structure of a membrane-impermeant antitumor sulfonamide complexed with isozyme II. J Med Chem 2005;48:5721–7. 10.1021/jm050333c 16134940

[11] a) Masini E, Carta F, Scozzafava A, Supuran CT. Antiglaucoma carbonic anhydrase inhibitors: a patent review. Expert Opin Ther Pat 2013;23:705–16. b) Fabrizi F, Mincione F, Somma T, et al A new approach to antiglaucoma drugs: carbonic anhydrase inhibitors with or without NO donating moieties. Mechanism of action and preliminary pharmacology. J Enzyme Inhib Med Chem 2012;27:138–47. c) Şentürk M, Gülçin İ, Beydemir Ş, et al In vitro inhibition of human carbonic anhydrase I and II isozymes with natural phenolic compounds. Chem Biol Drug Des 2011;77:494–9. d) Supuran CT, Mincione F, Scozzafava A, et al Carbonic anhydrase inhibitors—part 52. Metal complexes of heterocyclic sulfonamides: a new class of strong topical intraocular pressure-lowering agents in rabbits. Eur J Med Chem 1998;33:247–54.

[12] a) Aggarwal M, Kondeti B, McKenna R. Anticonvulsant/antiepileptic carbonic anhydrase inhibitors: a patent review. Expert Opin Ther Pat 2013;23:717–274. b) Di Fiore A, De Simone G, Alterio V, et al The anticonvulsant sulfamide JNJ-26990990 and its S, S-dioxide analog strongly inhibit carbonic anhydrases: solution and X-ray crystallographic studies. Org Biomol Chem 2016;14:4853–8. 10.1517/13543776.2013.782394 23514045

[13] a) Scozzafava A, Supuran CT, Carta F, Antiobesity carbonic anhydrase inhibitors: a literature and patent review. EOTP 2013;23:725–35. b) Supuran CT, Nicolae A, Popescu A. Carbonic anhydrase inhibitors. Part 35. Synthesis of Schiff bases derived from sulfanilamide and aromatic aldehydes: the first inhibitors with equally high affinity towards cytosolic and membrane-bound isozymes. Eur J Med Chem 1996;31:431–8. c) Pacchiano F, Aggarwal M, Avvaru BS, et al Selective hydrophobic pocket binding observed within the carbonic anhydrase II active site accommodate different 4-substituted-ureido-benzenesulfonamides and correlate to inhibitor potency. Chem Commun (Camb) 2010;46:8371–3. d) Tars K, Vullo D, Kazaks K, et al Sulfocoumarins (1,2-benzoxathiine-2,2-dioxides): a class of potent and isoform-selective inhibitors of tumor-associated carbonic anhydrases. J Med Chem 2013;56:293–300.

[14] a) Neri D, Supuran CT. Interfering with pH regulation in tumours as a therapeutic strategy. Nat. Rev Drug Discov 2011;10:767–77. b) Boyd NH, Walker K, Fried J, et al Addition of carbonic anhydrase 9 inhibitor SLC-0111 to temozolomide treatment delays glioblastoma growth in vivo. JCI Insight 2017;2:92928 c) Mastrolorenzo A, Rusconi S, Scozzafava A, et al Inhibitors of HIV-1 protease: current state of the art 10 years after their introduction. From antiretroviral drugs to antifungal, antibacterial and antitumor agents based on aspartic protease inhibitors. Curr Med Chem 2007;14:2734–48. d) Casey JR, Morgan PE, Vullo D, et al Carbonic anhydrase inhibitors. Design of selective, membrane-impermeant inhibitors targeting the human tumor-associated isozyme IX. J Med Chem 2004;47:2337–47. e) Garaj V, Puccetti L, Fasolis G, et al Carbonic anhydrase inhibitors: synthesis and inhibition of cytosolic/tumor-associated carbonic anhydrase isozymes I, II and IX with sulfonamides incorporating 1,2,4-triazine moieties. Bioorg Med Chem Lett 2004;14:5427–33. f) Supuran CT. Carbonic anhydrase inhibition and the management of hypoxic tumors. Metabolites 2017;7:E48.

[15] a) Supuran CT. Advances in structure-based drug discovery of carbonic anhydrase inhibitors. Expert Opin Drug Discov 2017;12:61–88. b) Dogne JM, Hanson J, Supuran C, Pratico D. Coxibs and cardiovascular side-effects: from light to shadow. Curr Pharm Des 2006;12:971–5. c) Supuran Carbon- versus sulphur-based zinc binding groups for carbonic anhydrase inhibitors? J Enzyme Inhib Med Chem 2018;33:485–95. d) Kusuzaki K, Matsubara T, Murata H, et al Natural extracellular nanovesicles and photodynamic molecules: Is there a future for drug delivery? J Enzyme Inhib Med Chem 2017;32:908–16. e) Ward C, Langdon SP, Mullen P, et al New strategies for targeting the hypoxic tumour microenvironment in breast cancer. Cancer Treat Rev 2013;39:171–9.

[16] Canto de Souza L, Provensi G, Vullo D, et al Carbonic anhydrase activation enhances object recognition memory in mice through phosphorylation of the extracellular signal-regulated kinase in the cortex and the hippocampus. Neuropharmacology 2017;118:148–56. 28286213 10.1016/j.neuropharm.2017.03.009

[17] Kim CU, Song H, Avvaru BS, et al Tracking solvent and protein movement during CO_2_ release in carbonic anhydrase II crystals. Proc Natl Acad Sci USA 2016;113:5257–62. 27114542 10.1073/pnas.1520786113 PMC4868437

[18] Tu CK, Silverman DN, Forsman C, et al Role of histidine 64 in the catalytic mechanism of human carbonic anhydrase II studied with a site-specific mutant. Biochemistry 1989;28:7913–8. 2514797 10.1021/bi00445a054

[19] Briganti F, Mangani S, Orioli P, et al Carbonic anhydrase activators: X-ray crystallographic and spectroscopic investigations for the interaction of isozymes I and II with histamine. Biochemistry 1997;36:10384–92. 9265618 10.1021/bi970760v

[20] Huang S, Hainzl T, Grundström C, et al Structural studies of β-carbonic anhydrase from the green alga *Coccomyxa*: inhibitor complexes with anions and acetazolamide. PLoS One 2011;6:e28458. 22162771 10.1371/journal.pone.0028458 PMC3230598

[21] Tripp BC, Ferry JG. A structure-function study of a proton transport pathway in the gamma-class carbonic anhydrase from *Methanosarcina thermophila*. Biochemistry 2000;39:9232–40. 10924116 10.1021/bi0001877

[22] Tu C, Rowlett RS, Tripp BC, et al Chemical rescue of proton transfer in catalysis by carbonic anhydrases in the beta- and gamma-class. Biochemistry 2002;41:15429–35. 12484784 10.1021/bi026831u

[23] Temperini C, Scozzafava A, Vullo D, Supuran CT. Carbonic anhydrase activators. Activation of isozymes I, II, IV, VA, VII, and XIV with L- and D-histidine and crystallographic analysis of their adducts with isoform II: engineering proton-transfer processes within the active site of an enzyme. Chemistry 2006;12:7057–66. 16807956 10.1002/chem.200600159

[24] Temperini C, Scozzafava A, Vullo D, Supuran CT. Carbonic anhydrase activators. Activation of isoforms I, II, IV, VA, VII, and XIV with L- and D-phenylalanine and crystallographic analysis of their adducts with isozyme II: stereospecific recognition within the active site of an enzyme and its consequences for the drug design. J Med Chem 2006;49:3019–27. 16686544 10.1021/jm0603320

[25] Ilies M, Banciu MD, Ilies MA, et al Carbonic anhydrase activators: design of high affinity isozymes I, II, and IV activators, incorporating tri-/tetrasubstituted-pyridinium-azole moieties. J Med Chem 2002;45:504–10. 11784154 10.1021/jm011031n

[26] a) Supuran CT. carbonic anhydrase activators. Future Med Chem 2018;10:561–73. b) Draghici B, Vullo D, Akocak S, et al Ethylene bis-imidazoles are highly potent and selective activators for isozymes VA and VII of carbonic anhydrase, with a potential nootropic effect. Chem Commun (Camb) 2014;50:5980–3. 10.1039/c4cc02346c 24763985

[27] Scozzafava A, Supuran CT. Carbonic anhydrase activators: high affinity isozymes I, II, and IV activators, incorporating a beta-alanyl-histidine scaffold. J Med Chem 2002;45:284–91. 11784133 10.1021/jm010958k

[28] Angeli A, Del Prete S, Osman SM, et al Activation studies of the α- and β-carbonic anhydrases from the pathogenic bacterium *Vibrio cholerae* with amines and amino acids. J Enzyme Inhib Med Chem 2018;33:227–33. 29231751 10.1080/14756366.2017.1412316 PMC7012002

[29] Angeli A, Del Prete S, Donald WA, et al The γ-carbonic anhydrase from the pathogenic bacterium *Vibrio cholerae* is potently activated by amines and amino acids. Bioorg Chem 2018;77:1–5. 29316507 10.1016/j.bioorg.2018.01.003

[30] Angeli A, Del Prete S, Osman SM, et al Activation studies with amines and amino acids of the β-carbonic anhydrase encoded by the Rv3273 gene from the pathogenic bacterium *Mycobacterium tuberculosis*. J Enzyme Inhib Med Chem 2018;33:364–9. 29322836 10.1080/14756366.2017.1422250 PMC6009870

[31] Vullo D, Del Prete S, Osman SM, et al Comparison of the amine/amino acid activation profiles of the β- and γ-carbonic anhydrases from the pathogenic bacterium *Burkholderia pseudomallei*. J Enzyme Inhib Med Chem 2018;33:25–30. 29098887 10.1080/14756366.2017.1387544 PMC6009869

[32] a) Mollica A, Costante R, Akdemir A, et al Exploring new Probenecid-based carbonic anhydrase inhibitors: Synthesis, biological evaluation and docking studies. Bioorg. Med. Chem 2015;23:5311–8. b) Mollica A, Pinnen P, Stefanucci A, Costante R, The evolution of peptide synthesis: from early days to small molecular machines. Curr Bioact Comp 2013;9:184–202. c) Mollica A, Costante R, Novellino E, et al Design, synthesis and biological evaluation of two opioid agonist and Cav2.2 blocker multitarget ligands. Chem Biol Drug Des 2015;86:156–62.

[33] Khalifah RG. The carbon dioxide hydration activity of carbonic anhydrase. I. Stop-flow kinetic studies on the native human isoenzymes B and C. J Biol Chem 1971;246:2561–73. 4994926

[34] Ferraroni M, Del Prete S, Vullo D, et al Crystal structure and kinetic studies of a tetrameric type II β-carbonic anhydrase from the pathogenic bacterium *Vibrio cholerae*. Acta Crystallogr D Biol Crystallogr 2015;71:2449–56. 26627652 10.1107/S1399004715018635

[35] a) Akocak S, Lolak N, Vullo D, et al Synthesis and biological evaluation of histamine Schiff bases as carbonic anhydrase I, II, IV, VII, and IX activators. J Enzyme Inhib Med Chem 2017;32:1305–12. b) Angeli A, Vaiano F, Mari F, et al Psychoactive substances belonging to the amphetamine class potently activate brain carbonic anhydrase isoforms VA, VB, VII, and XII. J Enzyme Inhib Med Chem 2017;32:1253–9. c) Licsandru E, Tanc M, Kocsis I, et al A class of carbonic anhydrase I – selective activators. J Enzyme Inhib Med Chem 2017;32:37–46.

[36] a) Perfetto R, Del Prete S, Vullo D, et al Cloning, expression and purification of the α-carbonic anhydrase from the mantle of the Mediterranean mussel, Mytilus galloprovincialis. J Enzyme Inhib Med Chem 2017;32:1029–35. b) Abdoli M, Angeli A, Bozdag M, et al Synthesis and carbonic anhydrase I, II, VII, and IX inhibition studies with a series of benzo[d]thiazole-5- and 6-sulfonamides. J Enzyme Inhib Med Chem 2017;32:1071–8.

[37] a) Temperini C, Scozzafava A, Supuran CT. Carbonic anhydrase activation and the drug design. Curr Pharm Des 2008;14:708–15. b) Isik S, Guler OO, Kockar F, et al Saccharomyces cerevisiae β-carbonic anhydrase: inhibition and activation studies. Curr Pharm Des 2010;16:3327–36. c) Temperini C, Innocenti A, Scozzafava A, Supuran CT. Carbonic anhydrase activators: kinetic and X-ray crystallographic study for the interaction of D- and L-tryptophan with the mammalian isoforms I-XIV. Bioorg Med Chem 2008;16:8373–8. d) Temperini C, Innocenti A, Scozzafava A, et al. Carbonic anhydrase activators: L-Adrenaline plugs the active site entrance of isozyme II, activating better isoforms I, IV, VA, VII, and XIV. Bioorg Med Chem Lett 2007;17:628–35. e) Clare BW, Supuran CT. Carbonic anhydrase activators. 3: structure-activity correlations for a series of isozyme II activators. J Pharm Sci 1994;83:768–73.

[38] a) Temperini C, Scozzafava A, Puccetti L, Supuran CT. Carbonic anhydrase activators: X-ray crystal structure of the adduct of human isozyme II with L-histidine as a platform for the design of stronger activators. Bioorg Med Chem Lett 2005;15:5136–41. b) Temperini C, Scozzafava A, Supuran CT. Carbonic anhydrase activators: the first X-ray crystallographic study of an adduct of isoform I. Bioorg Med Chem Lett 2006;16:5152–6. c) Vullo D, Nishimori I, Innocenti A, et al Carbonic anhydrase activators: an activation study of the human mitochondrial isoforms VA and VB with amino acids and amines. Bioorg Med Chem Lett 2007;17:1336–40. d) Pastorekova S, Vullo D, Nishimori I, et al Carbonic anhydrase activators: activation of the human tumor-associated isozymes IX and XII with amino acids and amines. Bioorg Med Chem 2008;16:3530–6. e) Nishimori I, Onishi S, Vullo D, et al Carbonic anhydrase activators. The first activation study of the human secretory isoform VI. Bioorg Med Chem 2007;15:5351–7.

